# Supplementary material for: Recombination hotspots in soybean [Glycine max (L.) Merr.]
Source: G3 (Bethesda). 2023 Mar 31;13(6):jkad075. doi: 10.1093/g3journal/jkad075 (PMC10234384; doi:10.1093/g3journal/jkad075)
Supplement: jkad075_Supplementary_Data [file jkad075_supplementary_data.zip › Supplemental Material Legends.docx]

**SUPPLEMENTAL MATERIAL**

**Figures S1-S20.** **Recombination hotspots for each chromosome.** The two outer rings display the recombination rates in cM/Mbp (y-axis) for each biparental populations by physical distance along chromosomes, Williams 82 x PI479752 (blue) and Williams 82 x Essex (red). The inner most circle displays the significant hotspots for each population in their respective color.

**Table S1. Recombination hotspot location, size, and recombination rate in each population**

File S1. Genotype data for both recombinant inbred line populations.
